# Draft Genome Sequence and Annotation of the Apicomplexan Parasite *Besnoitia besnoiti*

**DOI:** 10.1128/genomeA.01200-17

**Published:** 2017-11-16

**Authors:** Gereon Schares, Pratap Venepally, Hernán A. Lorenzi

**Affiliations:** a Friedrich Loeffler Institute, Federal Research Institute for Animal Health, Institute of Epidemiology, Greifswald, Riems, Germany; b The J. Craig Venter Institute, Rockville, Maryland, USA

## Abstract

The apicomplexan parasite *Besnoitia besnoiti* is the causative agent of bovine besnoitiosis that affects livestock, particularly cattle. The definitive host of *B. besnoiti* is unknown and its transmission only partially understood. Here, we report the first draft genome sequence, assembly, and annotation of this parasite.

## GENOME ANNOUNCEMENT

Bovine besnoitiosis is reemerging (1) and has spread into several European countries recently. It is the cause of infertility or sterility in bulls, loss of weight, reduced milk production, and abortion in acutely infected dams. *Besnoitia besnoiti* tissue cysts are—in contrast to those of *Toxoplasma gondii* and *Neospora caninum*—surrounded by a characteristic secondary cyst wall and mainly located in the dermis, subcutis, fascia, the cardiovascular system, mucosa of the upper respiratory tract, conjunctiva, and, in males, the testes and epididymis. The genome sequence of *B. besnoiti* and its annotation are of great importance for understanding the parasite's biology, the development of vaccines and better diagnostic tools, and the phylogenetic separation from related parasites.

The sequenced clone, C6, of *B. besnoiti* strain Bb-Ger1 was initially isolated from a skin sample of a naturally infected bull in Germany in 2008 (2), grown in cell culture, and cloned by serial dilutions. After amplification of tachyzoites in cell culture, high-molecular-weight parasitic DNA was extracted. Genomic DNA was then size selected at 20 kbp before building a sequencing library using PacBio P6-C4 chemistry. The sequencing library was run in nine PacBio RS II single-molecule real-time (SMRT) cells to generate 1,352,628 sequencing reads that were processed and assembled with HGAP v3 (3). The resulting *B. besnoiti* genome assembly has 229 contigs with a mean coverage of 81×, an average reference consensus concordance of >0.999, and an *N*_50_ contig length of 4,079,493 bp.

To predict protein coding genes, a data set of 2,731 *T. gondii* proteins derived from gene models with full-length cDNA support, Pfam-A seed peptides, and the apicomplexan proteins from UniREF-100 (4) were aligned to the assembled *B. besnoiti* sequence with the splice-aware aligner software *nap* (5). Next, a subset of the UniREF-100 and *T. gondii* proteins that aligned with at least 70% coverage and 60% identity were independently aligned to the genome with GeneWise (6). In addition, we ran two gene prediction tools previously trained for *T. gondii*, GeneZilla (7), and two configurations of Augustus (8) using or not using GeneWise alignments as evidence. Prediction of the final gene set was carried out with Evidence Modeler software (9) by integrating gene predictions plus all protein alignments, with a higher weight assigned to GeneWise mappings.

Product names were assigned to protein coding genes in three steps. First, syntenic blocks between *B. besnoiti* and *T. gondii* were identified with DAGchainer (10), and *B. besnoiti* syntenic genes were assigned the product names from their *T. gondii* orthologues, as annotated in ToxoDB v34 (11). Next, *B. besnoiti* nonsyntenic genes were named based on their best reciprocal BLASTp match and then based on their best BLASTp hit (e-value < 1 × 10^−5^ and at least 30% coverage) against the apicomplexan UniREF-100 protein data set. Remaining *B. besnoiti* gene products were designated “hypothetical proteins.”

### Accession number(s).

This whole-genome shotgun project has been deposited in DDBJ/ENA/GenBank under the accession no. NWUJ00000000. The version described in this paper is the first version, NWUJ01000000.

## References

[1] European Food Safety Authority 2010 Bovine besnoitiosis: an emerging disease in Europe. Eur Food Saf J 8:1–15. http://www.efsa.europa.eu/en/efsajournal/pub/1499.

[2] Schares G, Basso W, Majzoub M, Cortes HC, Rostaher A, Selmair J, Hermanns W, Conraths FJ, Gollnick NS 2009 First in vitro isolation of *Besnoitia besnoiti* from chronically infected cattle in Germany. Vet Parasitol 163:315–322. doi:10.1016/j.vetpar.2009.04.033. 19477592

[3] Chin CS, Alexander DH, Marks P, Klammer AA, Drake J, Heiner C, Clum A, Copeland A, Huddleston J, Eichler EE, Turner SW, Korlach J 2013 Nonhybrid, finished microbial genome assemblies from long-read SMRT sequencing data. Nat Methods 10:563–569. doi:10.1038/nmeth.2474. 23644548

[4] Suzek BE, Wang Y, Huang H, McGarvey PB, Wu CH; UniProt Consortium 2015 UniRef clusters: a comprehensive and scalable alternative for improving sequence similarity searches. Bioinformatics 31:926–932. doi:10.1093/bioinformatics/btu739. 25398609 PMC4375400

[5] Huang X, Zhang J 1996 Methods for comparing a DNA sequence with a protein sequence. Comput Appl Biosci 12:497–506. doi:10.1093/bioinformatics/12.6.497. 9021268

[6] Birney E, Durbin R 2000 Using GeneWise in the *Drosophila* annotation experiment. Genome Res 10:547–548. doi:10.1101/gr.10.4.547. 10779496 PMC310858

[7] Allen JE, Majoros WH, Pertea M, Salzberg SL 2006 JIGSAW, GeneZilla, and GlimmerHMM: puzzling out the features of human genes in the ENCODE regions. Genome Biol 7:S9.1–S9.13. doi:10.1186/gb-2006-7-s1-s9. 16925843 PMC1810558

[8] Stanke M, Waack S 2003 Gene prediction with a hidden Markov model and a new intron submodel. Bioinformatics 19:ii215–ii225. doi:10.1093/bioinformatics/btg1080. 14534192

[9] Haas BJ, Salzberg SL, Zhu W, Pertea M, Allen JE, Orvis J, White O, Buell CR, Wortman JR 2008 Automated eukaryotic gene structure annotation using EVidenceModeler and the Program to Assemble Spliced Alignments. Genome Biol 9:R7. doi:10.1186/gb-2008-9-1-r7. 18190707 PMC2395244

[10] Haas BJ, Delcher AL, Wortman JR, Salzberg SL 2004 DAGchainer: a tool for mining segmental genome duplications and synteny. Bioinformatics 20:3643–3646. doi:10.1093/bioinformatics/bth397. 15247098

[11] Kissinger JC, Gajria B, Li L, Paulsen IT, Roos DS 2003 ToxoDB: accessing the *Toxoplasma gondii* genome. Nucleic Acids Res 31:234–236. doi:10.1093/nar/gkg072. 12519989 PMC165519

